# Factors contributing to rise in teenage pregnancy in Sekhukhune district, Limpopo province

**DOI:** 10.4102/curationis.v47i1.2482

**Published:** 2024-05-10

**Authors:** Ragosebo P. Sekopa, Patrone R. Risenga, Sheillah H. Mboweni

**Affiliations:** 1 Department of Health Limpopo, Polokwane, South Africa; 2 Department of Health Studies, College of Human Sciences, University of South Africa, Pretoria, South Africa

**Keywords:** factors, teenager, pregnancy, teenage pregnancy, exploring

## Abstract

**Background:**

The Department of Health in South Africa has reported an alarming total of 90 037 teenage girls between the ages of 10 years and 19 years who gave birth from March 2021 to April 2022, across all provinces and districts. The rise in teenage pregnancy is of serious concern as adolescents girls are more likely to experience difficult pregnancies and deliveries which could lead to detrimental effects on their health.

**Objectives:**

The study aimed to explore and describe factors contributing to the increase in teenage pregnancy in the Sekhukhune district of Limpopo.

**Method:**

The study was conducted in the healthcare facilities of Sekhukhune area. A qualitative, exploratory design was followed. Participants were purposively selected, and data were gathered through face-to-face individual interviews. Data analysis employed Tesch’s inductive, descriptive coding method.

**Results:**

Negligence, peer pressure, ambiguity, choice, lack of contraceptive use, and lack of family attachment were identified as exacerbating factors in the district’s surge in teenage pregnancy.

**Conclusion:**

To reduce teen pregnancy, it is crucial to promote contraception, enhance cooperation between schools and the government, involve families in sexual and reproductive health discussions, prioritise a supportive home environment, advocate for child support grants, revitalise school health services, and empower teenagers to make informed choices and resist peer pressure.

**Contribution:**

The study will provide guidance to policy makers and other stakeholders in developing appropriate programmes to address the problem and improve the health and socioeconomic status of adolescents in rural areas. This will reduce healthcare costs associated with complications and premature birth.

## Introduction

Pregnancy among teenagers is a serious issue worldwide. Early pregnancy frequently leads to serious health repercussions for teenagers (World Health Organization [WHO] 2022). These young adolescents giving birth to children suffer repercussions that have an impact on their well-being. Teenage mothers (those between the ages of 10 years and 19 years) had higher rates of eclampsia, puerperal endometritis, and systemic infections compared to women between the ages of 20 years and 24 years, and their offsprings have higher rates of low birth weight, premature delivery, and serious neonatal conditions. Pregnancy and childbirth have been identified as the greatest cause of death for females between the ages of 15 years and 19 years worldwide (WHO 2022). A total of 94% of maternal fatalities worldwide among women between the ages of 15 years and 49 years have been recorded in low- and middle-income countries (LMICs) (WHO 2022). Pregnancy among adolescents often causes medical and public health issues because it could severely impact the adolescent mother’s physical and social development, as well as their ability to reproduce (Papri et al. 2016).

According to Jonas (2022), South Africa saw a spike in the number of teenage pregnancies in some regions between 2018 and 2019, as well as more recently during the coronavirus disease 2019 (COVID-19) pandemic. These higher rates were partially caused by the COVID-19 shutdown, which made it much harder to obtain contraceptives (Jonas 2022). Although the current initiatives aimed at reducing adolescent pregnancies include educational programmes, community support, economic assistance, and policy interventions, they are still considered insufficient. (Nkhoma et al. 2020). According to the United Nations (UN 2020), in 34 nations, 29 of which are in Africa, the rate of adolescent fertility was 80 births or higher per 1000 adolescent girls between the ages of 15 years and 19 years in 2020. This rate was still quite high between 2015 and 2020. The rate was above 140 per 1000 for some of those 34 nations, including 6 in Africa, 4 in Latin America and the Caribbean, and 1 in Asia. A Canadian study examining risk factors and birth outcomes related to teenage pregnancy discovered that out of 25 263 pregnant women, 1 080 were adolescents aged 19 years or younger. In contrast, the fertility rate for women residing in low-income areas, aged 20–24 years, was 9%, while for those over 35 years,it was 11% (Wong & Seabrook, 2020). Indongo (2020) found that teenage pregnancy affects 20.4% of Namibian teenagers, a 4% rise from 2006 to 2007. Teenage pregnancy is more likely to be influenced by poverty as a primary contributing factor among adolescents from low-income households. In contrast, teenagers from wealthier households tend to experience lower rates of teenage pregnancy. According to a report by Laldas in 2018 on News 24, approximately 11% of global births occur among females aged 15 years to 19 years in low income households. A total of 95% of the births in this 11%, or almost all of them, occur in LMICs, including South Africa. To prevent teenage pregnancies and sexually transmitted infections (STIs), the South African government has developed a number of policies, including assuring access to contraception and developing a basic education strategy (Laldas 2018).

In South Africa, the *Children’s Act 38 of 2005*, chapter 8, section 134, subsection 2, specifies that:

Contraceptives other than condoms may be provided to a child on request by the child and without the consent of the parent or caregiver of the child if the child is at least 12 years of age. (p. 62)

In addition,

Proper medical advice is given to the child; and a medical examination is carried out on the child to determine whether there are any medical reasons why a specific contraceptive should not be provided to the child. (p. 62)

The Act further indicates in section 134, paragraph 3 that:

A child who obtains condoms, contraceptives or contraceptive advice in terms of this Act is entitled to confidentiality in this respect. (p. 62)

In order to provide guidance to officials, administrators, management teams of the schools, and educators in response to the pregnancies of the students, a National Policy was developed in 2018. Additionally, the policy tries to control and prevent student pregnancies in schools. The policy also addresses the high rate of pregnancy among students, as well as the familial and social context in which this occurs, and discusses options available to reduce unwanted and unintended pregnancies, how to manage prenatal and postnatal complications, how to limit stigma and discrimination associated with pregnancy, and most importantly, how to retain and re-enrol the affected student in school (Department of Basic Education 2018).

In the Sekhukhune district of Limpopo province, the Department of Health provides a range of initiatives to safeguard young individuals against STIs and unintended pregnancies. Various programmes, including awareness campaigns on HIV and AIDS transmission and Love Life, are made accessible to the community. These programmes encompass education, the provision of free condoms and contraception, as well as HIV counselling and testing services. However, despite these efforts, data obtained from the Fetakgomo Tubatse Local Municipality clinics indicate elevated rates of teenage pregnancy and STIs (Jonas 2022). The goal of the study is to comprehend the variables that have led to an increase in teen pregnancies in the Sekhukhune district of Limpopo province. The study used the Bioecological Model of Bronfenbrenner to explain and investigate the causes of the surge in teen pregnancies in Sekhukhune district. According to the model, a child’s development is determined by a complex system of relationships that is influenced by a variety of layers of the environment, ranging from the immediate family and educational settings to broader societal values, regulations, and practices (Guy-Evans 2020). Additionally, this hypothesis explains why we behave differently with family members than we do at work or school (Knapp et al. 2020).

## Research methods and design

### Study design

The qualitative approach, as well as the explorative, descriptive, and contextual designs, were used in this study to accomplish its objectives. Researchers made sure that all research methods were reliable and legitimate, and that any ethical issues with the study were resolved (Gray, Grove & Sutherland 2017; Pilot & Beck 2017).

### Study setting

The study was conducted in the Sekhukhune district municipality, one of Limpopo province’s most rural municipalities. This municipality is located in the northern region of South Africa, and is categorised as a category C municipality according to the 2011 census. It has a population of 1 076 840 and four local municipalities. A category C municipality has municipal executive and legislative authority over a region that includes other municipalities (Sekhukhune District Municipality 2023). The government has put retention strategies in place by providing a 12% allowance for scarce skills in this rural district, but human resources are still a problem. With 134 067 teenagers between the ages of 15 years and 19 years, which is 13.86% of the total population, this age group has the highest population (WHO 2022).

### Study population and sampling strategy

The research was carried out in three different healthcare facilities in the Sekhukhune area. These clinics were chosen based on their accessibility and the kind of services they provide, including those for acute and chronic illnesses, mother and childcare, and youth-friendly services. Pregnant teenagers aged 13–19 years who resided in the Sekhukhune district and used the clinics there for antenatal and mother-and-child health services made up the study’s sample. Purposive sampling was utilised to select 18 pregnant teenagers who participated in the study (Gray et al. 2017; Pilot & Beck 2017).

### Data collection

Following an explanation of the study’s goal and the signing of consent forms, the researcher began collecting data. Data were gathered using individual, semi-structured interviews with each participant. Face-to-face interviews were used to collect data because they have the benefit of allowing the researcher to assess the participant’s level of knowledge and cooperation as well as to acquire extra information through observation. Personal interviews, in the opinion of Holloway and Galvin (2017), enable the researcher to clarify any unclear questions as well as ask both open-ended and closed-ended questions. Interviews lasted 20 min to 30 min. A total number of 18 interviews were done, and the sample attained data saturation. Six pregnant teenagers were interviewed from each of the three selected healthcare facilities. Sepedi was used as the communal medium of communication because most of the Sekhukhune inhabitants speak this language. Participants ranged in age from 16 years to 19 years. Coronavirus disease 2019 guidelines were followed for gathering the data. To guarantee that the researcher could access all the data gathered during the interview, a digital recorder was employed. To document the participants’ verbal and nonverbal clues, field notes were taken.

### Data analysis

According to Pilot and Beck (2017), in qualitative data analysis, segments are joined together to form conceptual patterns that are meaningful. Additionally, it involves putting disparate pieces together to create insightful conceptual patterns. It also involves searching for general concepts and identifying prevailing ideas utilising an inductive methodology. To make sure that all data were accurately collected, the researcher started the data analysis process with a digital voice recorder. The goal of the analysis of the field notes and recordings was to look for words, phrases, descriptions, and terminology that are comparable to the study’s title. Because the interviews were held in Sepedi, the material obtained was recorded verbatim and later translated into English. The independent coder received both the verbatim transcriptions of the field notes and the field notes for separate data analysis. The researcher and the independent coder met to discuss and come to an agreement on the coded data after data analysis. Tesch’s inductive, descriptive coding method was used to analyse the data. Participants’ personal information and all study-related data were treated in strict confidence. On the laptop, every recording and exact transcript was saved with a strong password. For about 5 years or until the research goal has been achieved, a digital recorder, field notes, hard copies of transcripts, consent and assent forms, and COVID-19 screening instruments will be maintained behind locked doors. The information would not be accessible to anyone but those actively involved in data collection and analysis.

### Trustworthiness in qualitative studies

According to Brink, Van der Walt and Van Rensburg (2018), trustworthiness is evaluated as a means of assuring data quality in qualitative research. Transparency in all procedures to be followed during the study was used to ensure reliability. To enhance credibility, the researchers engaged in prolonged interactions with participants lasting between 20 min to 30 min. Member checking and peer debriefing were also employed to ensure that the data accurately reflected the participants’ experiences. Detailed descriptions of the study’s methodology, participants, and context were provided to enhance transferability. The researchers maintained an audit trail of the data analysis procedures and recorded data to ensure dependability. Confirmability was strengthened through the researchers’ reflexive journal, involvement in peer debriefing, and the inclusion of an external qualitative research expert to critically review the data analysis process (Creswell & Creswell 2018).

### Ethical considerations

Ethical clearance was obtained from the University of South Africa, College of Human Sciences Research Ethics Committee (CREC), with reference number 2020-CHS-60756349. Permission to conduct the study was obtained from the Department of Health Limpopo, primary healthcare office in Sekhukhune District and Provincial office, and operational managers of the three selected Sekhukhune clinics (Reference number is LP-2021-01-08). Parents or legal guardians gave their consent for learners under the age of 18 years to take part in the study. All participants provided consent (for minors under the age of 18 years) or informed consent (for minors over the age of 18 years), following a thorough description of the goals, advantages, and dangers of the study, including the use of the digital recorder. The interview was performed in a private space, and participants were given the assurance that the information would not be able to be related to them. Real names were not used, only codes were used. Assurance was given that participants’ personal information and all study-related data would be treated in strict confidence. Transcripts and digital recorders would be kept secure for about 5 years, or until the intended study goal had been achieved. The information would not be accessible to anyone but those who were directly involved in data collection and analysis.

## Results

### Characteristics of study participants

In total, 18 participants were interviewed. All the participants, ranging in age from 16 years to 19 years, had teenage pregnancies and are now mothers; the information offered includes their actual experiences. The participants’ ages, number of pregnancies, and educational levels are shown in Table 1.

**TABLE 1:** Characteristics of participants.

Participant no.	Age of participants (years)	Number of pregnancies	Level of education
1.	18	First pregnancy	Grade 11
2.	19	Second pregnancy	Grade 12
3.	19	First pregnancy	Grade 12
4.	19	First pregnancy	Post-matric
5.	19	First pregnancy	Failed Grade 12
6.	18	First pregnancy	Grade 11
7.	19	First pregnancy	Passed Grade 12
8.	19	First pregnancy	Passed Grade 12
9.	19	First pregnancy	Grade 12
10.	19	First pregnancy	Failed Grade 12
11.	19	First pregnancy	Passed Grade 12
12.	17	First pregnancy	Grade 12
13.	17	First pregnancy	Grade 11
14.	19	First pregnancy	College
15.	17	First pregnancy	Grade 10
16.	18	First pregnancy	Grade 11
17.	19	First pregnancy	Grade 11
18.	16	First pregnancy	Grade 9

### Themes

Four themes and 12 subthemes emerged from the data after analysis as represented in Table 2.

**TABLE 2:** Themes and subthemes.

Themes	Subthemes
THEME 1: Sources of teenage pregnancy information to teenagers	1.1 Family as a source of information 1.2 School and teenage pregnancy information 1.3 Health institutions and teenage pregnancy information
THEME 2: Recommendations to reduce teenage pregnancy	2.1 Teenagers to use contraceptives 2.2 School health services 2.3 Home environment and parental love 2.4 Governmental exclusion of child support grant to teenage mother
THEME 3: Aggravating factors in teenage pregnancy	3.1 Negligence and peer pressure 3.2 Lack of family affection 3.3 Uncertainty and choice
THEME 4: Support systems that helped teenagers to cope with teenage pregnancy	4.1 Family support 4.2 Support from partner

The following section discusses in detail the four themes and their subthemes. Each theme contains an outline and subthemes, followed by quotes. The study’s conclusions have been supported by a literature review.

#### Theme 1: Sources of teenage pregnancy information to teenagers

Participants pointed out many settings, including the family, school, and medical facilities, where information about teen pregnancies is disseminated and relayed to pregnant teenagers.

**Family as a source of information:** The participants indicated that their family was one of the sources of information they approached on how to take care of themselves while expecting:

‘My aunt offers me suggestions. They are incredibly helpful to me because I have a stress-free, healthy lifestyle.’ (P1, 18-year-old, female)

‘My parents provide me with pregnancy-related information, including advice on what to do, how to act, and what to eat. Since I became pregnant, I have not been ill, therefore the knowledge is crucial.’ (P7, 19-year-old, female)

**School and teenage pregnancy information:** Participants who attended school while pregnant noted that they obtained advice on how to behave properly as a pregnant teenager and that this advice was valuable. The quotes below support this notion:

‘We were counselled against becoming pregnant at a young age in school. The information is useful since, when we are young, we become pregnant and burden our parents.’ (P6, 18-year-old, female)

‘At school, we learned a lot of lessons about teen pregnancies. I now understand how to look for myself and avoid getting pregnant again.’ (P9, 19-year-old, female)

‘In class. Our school occasionally receives visits from nurses who inform us about pregnancy. The knowledge is crucial because I can use it to counsel other young girls against getting pregnant at an early age.’ (P12, 17-year-old, female)

**Health institutions and teenage pregnancy information:** Some of the participants who made appointments for antenatal care in medical facilities said they received information about pregnancy through the Mom-Connect programme. This subtheme is supported by the quotes below:

‘Mom Connect linked me at the clinic. I can better understand the phases of pregnancy and how to care for you as a pregnant woman thanks to this knowledge. These words are beneficial to other adolescent mothers-to-be in addition to me.’ (P5, 19-year-old, female)

‘At the clinic, they warned me not to eat dirt and not to drink cold water because I would experience complications when giving birth to my child. In order for me to have a healthy child, they also advised me to eat well.’ (P11, 19-year-old, female)

‘At the clinic, on Google and social media. They are crucial because they impart knowledge about pregnancy and how to act like a pregnant mother.’ (P16, 18-year-old, female)

#### Theme 2: Recommendations to reduce teenage pregnancy

Participants suggested that various measures be taken by teens, schools, parents, and the government to prevent teenage pregnancy. As listed below, this theme has four subthemes.

**Teenagers to use contraceptives:** Most participants admitted that teenage use of contraception will aid in reducing teen pregnancy. However, only a condom and an injection were specifically mentioned, while the other methods were not. The quotes below support the subject:

‘Young girls should look after themselves and go to the doctor frequently. They must use condoms and adhere to condom usage guidelines. They must resist peer pressure, lack of parental guidance, and other factors that prevent them from starting a family.’ (P4, 19-year-old, female)

‘Teenagers should attend clinics frequently, pay attention to their parents’ advice, and take precautions to avoid getting pregnant.’ (P9, 19-year-old, female)

‘Whether or not they are sexually active, young girls should be encouraged to frequent clinics and acquire contraceptives starting at age 12.’ (P17, 19-year-old, female)

**School health services:** Participants stressed the possibility that having access to school health services could reduce teen pregnancy. The suggested approaches include having nurses come into schools on specific days to give teenagers contraceptives or having clinics located within schools. Participants also mentioned that primary schools should provide lessons on teen pregnancy. The quotes that follow fit with the subtheme:

‘Nurses should go to schools to distribute contraceptives and educate students about teenage pregnancy and condoms.’ (P2, 19-year-old, female)

‘In order to provide health advice and, if possible, to provide contraceptives in front of parents, the Department of Health should visit schools, particularly primary schools, as well as welcome parents to the facilities.’ (P5, 19-year-old, female)

‘Since clinics are far from some communities, it will be simple for children to access health services if schools can have them on the school grounds.’ (P7, 19-year-old, female)

‘Pregnancy education must be taught in primary schools because students are affected since they do not comprehend this type of education, which begins in grade 10. Therefore, if this education can begin in primary schools, children will be able to ask their parents about what they have been taught in school, which would increase understanding.’ (P10, 19-year-old, female)

**Home environment and parental love:** Two participants suggested that a loving and supportive home environment for teenagers would also be highly helpful in reducing teenage pregnancies. The quotes that follow support the subtheme:

‘Teenage pregnancy will decrease if family issues can be resolved, allowing parents to provide their kids with the support and love they need, as this will prevent them from leaving the house to find other people to love them.’ (P3, 19-year-old, female)

‘Home environment can be used to reduce teenage pregnancy, along with parental support and love for teenagers as family members.’ (P1, 18-year-old, female)

**Governmental exclusion of child support grant to teenage mothers:** Participants also suggested that withholding child support grants from teenage mothers can be used as a strategy to decrease teen pregnancy. The quote below supports this theme:

‘Maybe if the government can tell teenagers between 13–19 years who fall pregnant, they will not get child support grants because some of us fall pregnant because we need child support grants.’ (P8, 19-year-old, female)

#### Theme 3: Aggravating factors in teenage pregnancy

The participants discussed aggravating elements that contribute to teen pregnancy, such as irresponsibility, peer pressure, unpredictability, choice, non-use of contraception, and lack of parental love. Three subthemes make up this theme.

**Negligence and peer pressure:** Participants mentioned that pressure and carelessness are factors that make teenage pregnancy worse. In terms of carelessness, the following were mentioned: failure to use protection such as condoms, missing the date of the contraceptive injection as well as ceasing the use of contraception, the thrill of engaging in sexual activity, and disregarding parental advice about having sex when you are a teenager. The quotes listed below support this theme:

‘Carelessness and disregard for detail. I heard about condoms and contraceptives, but I never had time to visit the clinic because I was constantly in class or working on schoolwork.’ (P2, 19-year-old, female)

‘The teenager was trembling while gazing at her feet and fidgeting with her fingers. “I was chasing boys without using any defense.”’ (P6, 18-year-old, female)

‘I got carried away with my feelings and indulged in sexual activity without using any protection because I was excited and taken by them.’ (P12, 17-year-old, female)

‘Peer influence. Because my buddy was expecting and is now a mother, I did become pregnant.’ (P13, 17-year-old, female)

‘I was not listening to my mother when she advised me and told me not to go out with boys especially at night. I was using contraceptives sometimes.’ (P17, 19-year-old, female)

‘I did not use contraceptives. I stopped using contraceptives in 2018 because of continuous vaginal bleeding whenever I use contraceptives.’ (P1, 18-year-old, female)

**Lack of family affection:** One participant claimed that the absence of motherly love or other forms of family affection led to her getting pregnant because she left her house to live with her boyfriend in search of love that was no longer present at home. All the mother’s love for her children was transferred to the new stepdad when he joined the household. The quote below supports this subtheme:

‘When you don’t feel loved by your family, you go out looking for love elsewhere. My mother married my stepfather in 2019 and brought him home, which caused everything in our home to alter. As a result, I ran away from home to live with my boyfriend, with whom I feel more loved than I do with my family. When responding to this query, she was hunched over and appeared dejected.’ (P13, 19-year-old, female)

**Uncertainty and choice:** Three participants had conflicting emotions about becoming pregnant; the first said she wanted a child but did not feel ready, thus her boyfriend’s suggestion of becoming pregnant was something she did not agree with. The second woman hinted that they were using condom at the time. She was therefore unsure of what had happened because she fell pregnant despite using protection. The final participant became pregnant because she wanted to have a child. See the quotes below:

‘I wanted to have a baby but also felt that I am not ready to have a child. It was my boyfriend’s idea which I did not support at the beginning.’ (P4, 19-year-old, female)

‘I do not know what happened because I was always using condoms when I indulged in sexual activity.’ (P5, 19-year-old, female)

‘“I fell pregnant because I wanted to have a child”. [*She was busy mumbling and signalling with her hands saying that she had already answered the question*].’ (P11, 19-year-old, female)

#### Theme 4: Support systems that helped teenagers to cope with teenage pregnancy

Participants described how their family and romantic partners provided them with support in a way that allowed them to survive their teenage pregnancies. According to the details below, this theme had two subthemes.

**Family support:** Most participants said that having support from their mothers, grandparents, aunts, and friends made it easier for them to continue with their pregnancies. The following quotes support the subtheme:

‘Yes, my grandma and parents. Every time my grandmother receives an SASSA award, she sends me money. They offer me emotional support and motivate me to study because I’m in matric now and want to pass it and find employment so that I can support my kids.’ (P2, 19-year-old, female)

‘Yes, my mother supports me. They inquire about my medicine intake every time. When I need money, they help me out and constantly check to see how I’m doing.’ (P4, 19-year-old, female)

‘Yes, my mother and friends told me to end things with him and concentrate on looking after myself and reducing stress.’ (P18, 16-year-old, female)

**Support from partner:** One of the pillars that assisted youngsters to survive teenage pregnancy as mentioned by the participants was support from partners. The following quotes support this subtheme:

‘Yes, my boyfriend helped me emotionally and pushed me to complete my schoolwork because I’m in matric and want to finish it and find employment so that I can support my kids.’ (P2, 19-year-old, female)

‘Yes, my partner. My partner is paying for my quarter-room apartment, where I currently reside. When I go to school, he also buys meals and gives me money. When discussing her guy, she was grinning.’ (P3, 19-year-old, female)

‘Yes, my boyfriend supports me. He always checks to see if I’ve taken my medicine correctly. And he always checks to see whether I’m alright.’ (P4, 19-year-old, female)

## Discussion

### Sources of teenage pregnancy information to teenagers

According to the study findings, teenagers who are pregnant are more likely to get information on teen pregnancies from family, as well as at school and health facilities. The study by Skosana and Mogale (2020) on the disconnections and exclusions of parents in the prevention of teenage pregnancy lends validity to these findings. The results show that parents are seen as the key sexual educators who must communicate sexual information in an accepting manner. The article continued by stating that parents play a crucial role in the sexual socialisation of kids and teenagers, and that information and messages that are not shared between parents and kids can affect how kids and teenagers make sexual decisions (Skosana & Mogale 2020). A study conducted by Skinner et al. (2018) affirms the advantages of the Mom-Connect programme for pregnant mothers, as respondents attest that it had empowered them with the knowledge to manage their pregnancy, and enhanced their child-rearing skills, including information about their own health and caring for the newborn. It is important to take your teenager to annual wellness appointments and to give her the opportunity to speak with the doctor alone. In this regard, Morin (2021) pointed out that, sometimes, teens are hesitant to ask their parents about sex or contraceptives but are more comfortable discussing these topics with medical professionals, counsellors, and other trusted adults. This finding confirms the current study’s findings that health facilities are a reliable source of information on teenage pregnancies. In this study, it was shown that moms, aunts, grandparents, all had a significant part in assisting teenagers who were pregnant by sharing knowledge on how pregnant people should care for themselves.

Teenagers who maintained their education while pregnant claimed to have received more information on teenage pregnancy in the school setting. Teenagers who visited medical facilities to schedule antenatal care services had access to pregnancy information through Mom-Connect programme. Teenagers who were pregnant regarded all the information they obtained from various sources as being of utmost importance. Teenagers offered a number of suggestions that may be used to combat teenage pregnancy, which is the subject of the following theme.

### Strategies suggested by teenagers to reduce teenage pregnancy

In this study’s findings, various government, home, and school initiatives to lower teenage pregnancies were suggested. Participants stressed the need for parental support for teenagers and involvement in sex education. Teenagers’ use of contraception was emphasised. It was also recommended that the government and schools be involved.

These findings are reinforced by research conducted by Mann, Bateson and Black (2020), which found that managing and preventing teen pregnancy require extensive initiatives, including the community, healthcare providers, and schools. A study by Qolesa (2017) found that presenting seminars at schools to encourage parent-child communication is urgently needed. The Departments of Health, Social Development, and Education may be able to help with this. This intersectoral strategy aims to empower and encourage parents and learners to develop effective communication skills so they can cooperate to address issues that teenagers face, such as sexual and reproductive health.

The United Nations International Children’s Emergency Fund (UNICEF 2018) emphasised the importance of parents educating their children about sexual and reproductive health. It also stressed that teaching children about sex at a young age was not frowned upon, and acted as a safeguard against precocious sexual behaviour that might result in teenage pregnancy (Budiharjo, Theresia & Widyasia 2018). Participants also suggested that withholding child support grants from teenage moms could be used as one of several strategies to decrease teen pregnancy. Mahamotsa (2018) suggested that there must be a policy regarding regulation of the number of children to benefit from the child support grant, as this will deter teenage girls from giving birth or having babies with the hope of getting more money. This denotes that doing away with child support might gradually contribute positively to the reduction of teenage pregnancy; however, this is contrary to strategies that government has in dealing with poverty.

### Aggravating factors in teenage pregnancy

The findings of this study showed several reasons that make teenage pregnancy more likely, including irresponsibility, peer pressure, unpredictability, choice, non-use of contraception, and lack of parental love. Participants admitted to having teenage pregnancies as a result of the aforementioned circumstances. These conclusions were corroborated by the findings of a study by Maemeko, Nkengbeza and Chokomosi (2018), which showed that strained parental relationships and a negative environment in their homes encouraged teenagers to seek love and attention from their male peers, which often leads to them engaging in sexual behaviour that may ultimately result in teenage pregnancy. Carelessness in sexual behaviours that often occurs among young people was one of the concerns highlighted as contributing to an increase in adolescent pregnancies. Dissatisfactory interaction between parents and their teenage children as well as drug and alcohol abuse were also mentioned (Maemeko et al. 2018).

Peer pressure, sexual abuse, alcohol and drug usage, and the influence of the media have all been identified as significant reasons for teenage pregnancy in rural Eastern Uganda, according to Nadkarni et al. (2022). According to Wagle’s (2019) research, teenage pregnancy is caused by a variety of factors, including parental neglect, a lack of formal and informal education, a lack of sex education, a lack of parental communication and supervision, peer pressure, sexual abuse, drug abuse, a history of forced marriage, a desire for children, a lack of knowledge about contraceptives, and a lack of school fees. Other contributing factors include poverty and peer pressure. The studies mentioned above support the study’s conclusions about aggravating factors, including poverty, a lack of contraception, and peer pressure.

### Support systems that helped teenagers to cope with teenage pregnancy

Participants in this study disclosed that the assistance of their family and romantic partners had enabled them to survive the teenage pregnancy. Kirchengast (2016) noted that the prevalence of teen pregnancies has given rise to alarm. According to the survey, young girls experiencing teenage pregnancies are getting more support and motivation to advance in life from family, friends, and total strangers, dispelling myths and exposing challenges related to adolescent pregnancy. According to Govender, Naidoo and Taylor (2020), some teenage moms receive financial and emotional assistance from their partners, biological mothers, and grandmothers. The study shows that some adolescent mothers went to live with their boyfriends after disclosing their pregnancy status, having been rejected by their family members, some also refusing to support them emotionally and financially (Govender et al. 2020). According to Duncan (2019), it is important for parents to support their pregnant teenager by reassuring her of their unconditional love and concern, and also offering to tell close family members or friends about the pregnancy. This is consistent with the study’s findings, which showed that individuals had support from their partners and families.

### Recommendations

Government should ensure that more budget is allocated to the Department of Health in Limpopo to allow the Department to purchase contraceptives from the manufacturer and deliver them on time to the health facilities. More budgets will also allow the Department of Health to construct few clinics inside school grounds that will be staffed by medical professionals and furnished with medical supplies so that schoolchildren can obtain medical care. The National Adolescent-Friendly Clinic Initiative (NAFCI) should be followed by all primary healthcare (PHC) facilities. Parents should be encouraged to support their teenagers and participate in sex education. For teenagers to be able to comprehend their bodies, they should be taught about sexual and reproductive health, starting at age 12.

## Conclusions

The article describes the factors that are leading to the surge in teenage pregnancy in Sekhukhune district. Carelessness, peer pressure, uncertainty, choice, lack of contraception use, and a lack of family attachment were identified as exacerbating factors in the increase in teenage pregnancy in the Sekhukhune district. Teenage pregnancy can be reduced if schools, parents and government work together. Parental involvement in sex education and support for teenagers, the use of contraception, and the involvement of school and government in the campaign to minimise teenage pregnancy should all be emphasised.
